# Penile self-amputation due to cannabis-induced psychosis: a case report

**DOI:** 10.1186/s13256-022-03267-0

**Published:** 2022-01-30

**Authors:** Nantanan Jengsuebsant, Sirapat Benjachaya, Jaraspong Vuthiwong, Theerapon Tangsuwanaruk

**Affiliations:** 1 Department of Emergency Medicine, Faculty of Medicine, Chiang Mai University, 110 Inthawaroros road, Sribhumi, Amphoe Muang Chiang Mai, Chiang Mai, 50200 Thailand; 2 Division of Urology, Department of Surgery, Faculty of Medicine, Chiang Mai University, Chiang Mai, 50200 Thailand

**Keywords:** Cannabis, Case report, Penile self-amputation, Psychosis, Self-mutilation

## Abstract

**Background:**

In recent decades, cannabis has been widely used around the world for medical and recreational purposes, both legally and illegally. Aside from its therapeutic benefits, cannabis exhibits many adverse effects. Psychosis is one of the potentially harmful effects of cannabis.

**Case presentation:**

A 23-year-old Thai man, who reported cannabis use for 2 years and discontinued for 3 months, restarted smoking two bongs (2 g equivalence) of cannabis. Two hours later, he had a penile erection, felt a severe persistent sharp pain in his penis, and reported that his glans looked distorted. Intending to eradicate the pain, he decided to trim the penile skin several times and completely amputated his penis himself using scissors. Cannabis-induced psychosis was diagnosed because symptoms began after cannabis use, without evidence of other substance abuse. To confirm the cannabis exposure, his urine immunoassay was positive for delta-9-tetrahydrocannabinol (Δ^9^-THC). The distal penis was deemed too dirty and fragile for reconstruction. Bleeding was controlled, penile stump irrigated and debrided, and scrotal urethrostomy was performed by a urologist. After admission and cannabis discontinuation, his delusion and hallucination subsided.

**Conclusions:**

Cannabis-induced psychosis is an adverse effect of cannabis, which may lead to impaired judgement unexpected self-harm. A multidisciplinary team approach, including a primary care physician, an emergency physician, a urologist, and a psychiatrist, is essential when dealing with a patient with cannabis-induced psychosis and a urogenital injury.

## Background

In recent decades, cannabis, dried–grated flowers and leaves of *Cannabis sativa*, has been widely used for medical and recreational purposes [1]. Aside from its therapeutic benefits, cannabis exhibits many adverse effects, including impaired judgement. With heavy use, paranoia and psychosis may be expected [2, 3]. Male genital self-mutilation from psychiatric disorder or substance-induced psychosis have been reported, however, the exact prevalence of these conditions is unknown. Some reports state about hundred cases within the past two decades [4, 5]. However, self-amputation of penis in cannabis-induced psychosis has rarely been reported [6]. In this case report, we highlight a psychotic condition induced by recreational cannabis use, leading to penile self-amputation in a different manner.

## Case presentation

**Fig. 1:**
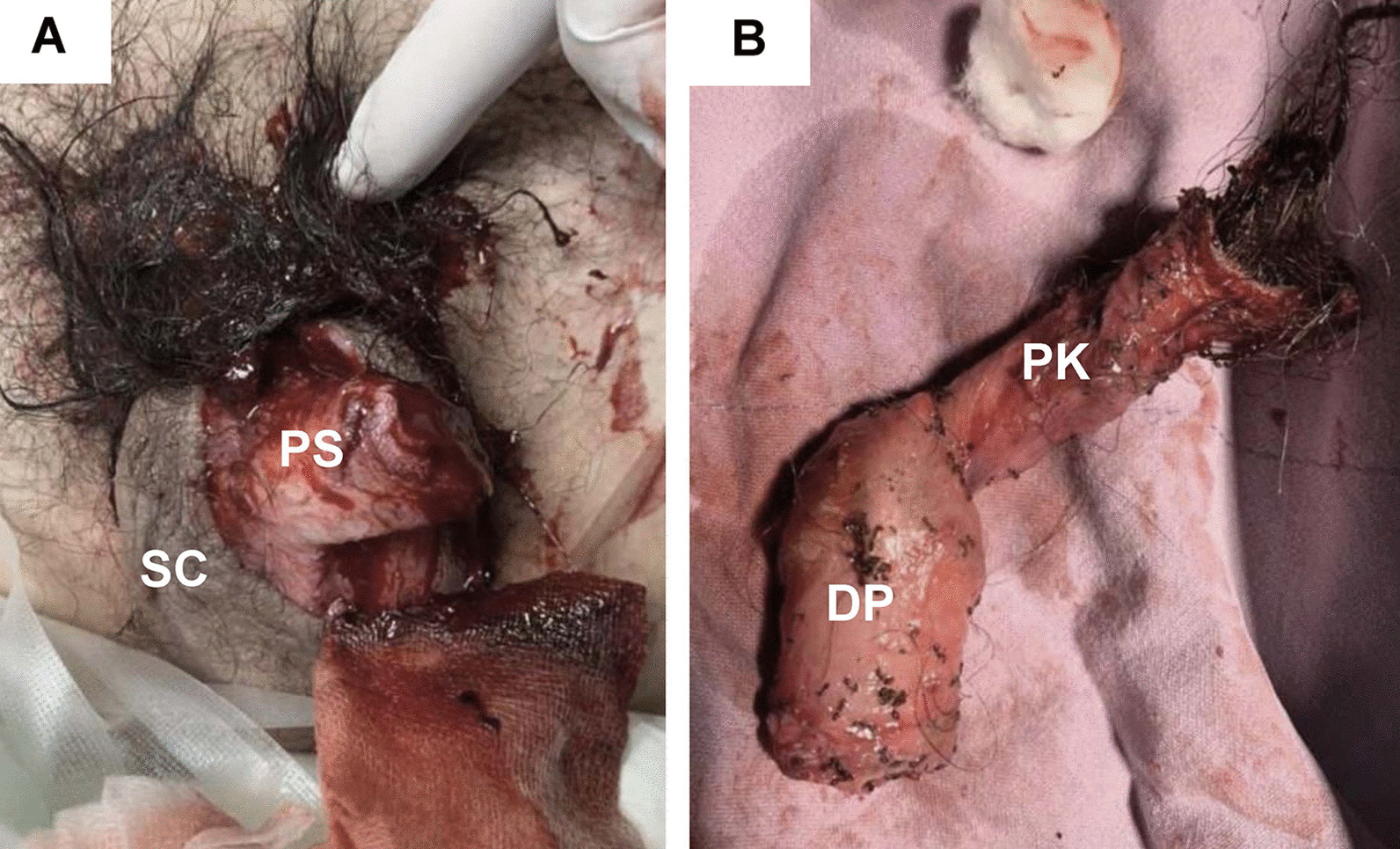
**A** The remaining penile stump. **B** The amputated distal penis. *DP* amputated distal penis, *PS* penile stump, *PK* penile skin, *SC* scrotum

A 23-year-old Thai man, who reported cannabis use for 2 years and discontinued for 3 months, restarted smoking two bongs (2 g equivalence) of cannabis. Besides cannabis, he reported drinking five cups of coffee per day. He denied depressed mood or manic symptoms, alcohol consumption, other substance abuse, or previous self-harm. He denied any underlying disease, previous psychiatric treatment, nor a family history of psychiatric condition. Two hours later, he had a penile erection without sexual stimulation, felt a severe persistent sharp pain in his penis, and reported that his glans looked distorted. Intending to eradicate the pain, without command hallucination, he decided to trim the penile skin several times and completely amputated his penis himself using scissors. He reported awareness throughout the process. After 2 hours, the bleeding had not stopped. He was brought to a primary care hospital, where he was given intravenous cloxacillin, tetanus prophylaxis, and referred to our emergency department. On arrival, he was hemodynamically stable and cooperative. There was active bleeding at the penile base and a 5-cm lacerated wound at the scrotum. The remaining penile stump was 2 cm in length with loss of the whole penile skin. The amputated distal part of the penis was contaminated with ants and had fragile dorsal veins (Fig. 1). Urine immunoassay was positive for delta-9-tetrahydrocannabinol (Δ^9^-THC).

A urologist was consulted for surgical intervention. The distal penis was deemed too dirty and fragile for reconstruction. The patient was transferred to the operating theater for emergency surgery. Bleeding was controlled, the penile stump irrigated and debrided, and scrotal urethrostomy was performed. He was admitted to the surgical ward. A psychiatrist diagnosed the patient with substance-induced psychotic disorder. His mental status examinations found he had visual and auditory hallucinations, such as seeing moving shadows, hearing birds chirping or insects buzzing, depressed mood, and restricted affect. He was coherent and delusional, with no suicidal ideas. Supportive psychotherapy and 2 mg/day of risperidone were initiated. After admission and cannabis discontinuation, his delusions and hallucinations subsided. He stayed in the hospital for 14 days. The dosage of risperidone was adjusted to 6 mg/day at discharge. The brief psychiatric rating scale (BPRS) showed a score of 28 before treatment compared with a score of 18 after treatment. After 2 weeks, the patient was able to void in a sitting position, without wound infection. He denied visual or auditory hallucinations. Second stage penoplasty with a scrotal flap was planned, however, the patient was not available for follow-up and further management as he had relocated.

## Discussion and conclusions

In this patient, the diagnosis of substance-induced psychotic disorder could be made as his symptoms began after cannabis use, without evidence of other substance abuse, and his urine immunoassay was positive for delta-9-tetrahydrocannabinol (Δ^9^-THC), as well as from the resolution of psychotic symptoms within 4 weeks after abstinence [7]. To date, the 2019 Annual Report of the American Association of Poison Control Centers reported that patients using cannabinoids and analogues account for 0.8% of fatalities among all substance-exposed fatalities [8]. The Oregon/Alaska Poison Center also reported that routes of cannabis exposure were ingestion (73.9%), inhalation (22.5%), topical/parenteral/rectal (0.8%), and unknown/other (2.8%) between 2015 and 2017. Most of the patients were male, and two-thirds among the overall age group were intentional use [9].

Cannabis use was reported to increase the risk of psychosis, loss of insight, and thought disorder leading to unexpected behavior, including in patients with no previous psychiatric disorders, as seen in our patient [10–12]. The severity of psychosis depends on the amount of THC [13]. THC, consumed by smoking cannabis, is one of the primary cannabinoids producing psychoactive effects through the dopaminergic pathway [14]. Over time, the concentration of THC in illegal cannabis samples has increased from less than 4% to more than 12%, suggesting a need for stricter regulation as a higher THC concentration is associated with more adverse effects [2].

Self-amputation of the penis due to cannabis-induced psychosis, as in our patient, is a devastating event that interferes with the quality of life, such as urination dysfunction or sexual function. Although psychosis is a manifestation in cannabis users, the method of self-amputation of the penis in cannabis-induced psychosis varies. Khan *et al.* reported a case of self-amputation of the penis in a patient with cannabis-induced psychosis whose penis was chopped off by a sharp object [6]. On the other hand, in our patient, the penile skin was trimmed several times and then completely amputated using scissors. Several times of trimming rather than stopping after the first trimming indicated the calm self-harm and persistence due to psychosis. After self-amputation in our patient who denied previous personal hygiene neglect and antisocial behavior, the amputated distal part of the penis was contaminated with ants. Although self-neglect is a finding in some substances such as methamphetamine [15], we could not determine if discarding of the amputated part of his penis was related to a cannabinoid effect leading to self-neglect or his intention to eradicate the origin of pain at the penis.

From our patient’s history of penile erection with persistent sharp pain, priapism could also be suspected in our patient. Priapism is a condition where the penis remains erect for at least 4 hours, without sexual stimulation [16]. However, our patient’s condition did not fulfill priapism diagnostic criteria because the penis was cut off before the erection exceeded 4 hours. Sickle cell disease as a priapism risk factor is a rare disease in our patient population, and his blood investigations did not demonstrate anemia [17]. Although there are some previous case reports about cannabis use and priapism, the reported patients used cannabis combined with other substances. Evans *et al.* reported concurrent cannabis, steroid, and cocaine use in an insulin-dependent diabetes mellitus patient [18]. Tran *et al.* reported a patient with priapism after use cannabis and ecstasy. It could be the interaction between cannabis and ecstasy via ecstasy stimulating dopamine release in the brain. Synergistic interactions between ecstasy and cannabis might be possible [19]. In an animal model, dopamine receptor agonist increases central oxytocinergic neurotransmission and facilitates penile erection [20]. Therefore, cannabinoid use promoting dopaminergic pathway might play a role in penile erection [19]. Moreover, cannabinoids block the thoracolumbar sympathetic pathway, which could result in the penis being unable to detumescence and increasing the risk of priapism [17, 21]. THC interacts with a cannabinoid type 1 (CB1) receptor in the central nervous system (CNS), peripheral nervous system, and vasculature. Consequently, cannabinoids might potentiate vascular effects and lead to penile erection and priapism [17, 19]. Although SR 141716A is a CB1 receptor antagonist, it increases the glutamic acid and also activates the oxytocinergic neurons, leading to penile erection in the rat model [22]. Recently, a previous case report suggested a relationship between cannabis use alone and priapism. However, the patient smoked cannabis for the previous 6 months and no self-harm or psychosis occurred [17]. Although priapism is a painful event, self-amputation is rare in a patient with normal judgement. Thus, self-harm of our patient could be the effect of psychosis.

Acute cannabis exposure has been shown to have the following effects: CNS excitation (38.3%), CNS depression (24.4%), cardiac problems (14.6%), nausea and vomiting (9.5%), unusual/unexpected subjective sensation (strange, weird, bizarre) (3.6%), abdominal pain (2.4%), and psychosis (1.6%) [9]. Our patient also felt a severe persistent sharp pain in the penis after cannabis exposure. It might be an unusual/unexpected subjective sensation from the cannabis effect. However, we could not conclude that our patient’s sharp pain was because of priapism or an unusual/unexpected subjective sensation from cannabis exposure.

In summary, cannabis-induced psychosis is an adverse effect of cannabis, which may lead to impaired judgement and unexpected self-harm. A multidisciplinary team approach, including a primary care physician, an emergency physician, a urologist, and a psychiatrist, is essential when dealing with a patient with cannabis-induced psychosis and a urogenital injury.

## Data Availability

Data sharing is not applicable to this article as no datasets were generated or analysed during the current study.

## Abbreviations

BPRS: Brief psychiatric rating scale
CB1 receptor: Cannabinoid type 1 receptor
CNS: Central nervous system
ED: Emergency department
THC: Tetrahydrocannabinol

## References

[1] Hasin DS. US epidemiology of cannabis use and associated problems. Neuropsychopharmacology. 2018;43(1):195–212. 10.1038/npp.2017.198 PMC5719106 28853439

[2] Volkow ND, Baler RD, Compton WM, Weiss SRB. Adverse health effects of marijuana use. N Engl J Med. 2014;370(23):2219–27. 10.1056/NEJMra1402309 PMC4827335 24897085

[3] Caspi A, Moffitt TE, Cannon M, McClay J, Murray R, Harrington H, *et al*. Moderation of the effect of adolescent-onset cannabis use on adult psychosis by a functional polymorphism in the catechol-O-methyltransferase gene: longitudinal evidence of a gene X environment interaction. Biol Psychiatry. 2005;57(10):1117–27. 10.1016/j.biopsych.2005.01.026 15866551

[4] Raheem OA, Mirheydar HS, Patel ND, Patel SH, Suliman A, Buckley JC. Surgical management of traumatic penile amputation: a case report and review of the world literature. Sex Med. 2015;3(1):49–53. 10.1002/sm2.54 PMC4380914 25844175

[5] Mumoli N, Giorgi-Pierfranceschi M, Porta C, Manzionna G, Barberio M. Penile self-amputation. Intern Emerg Med. 2018;13(7):1133. 10.1007/s11739-018-1899-6 29943166

[6] Khan Mohd K, Usmani MA, Hanif SA. A case of self amputation of penis by cannabis induced psychosis. J Forensic Leg Med. 2012;19(6):355–7. 10.1016/j.jflm.2012.02.023 22847056

[7] American Psychiatric Association. Diagnostic and statistical manual of mental disorders: DSM-5. 5th ed. Arlington: American Psychiatric Association; 2013.

[8] Gummin DD, Mowry JB, Beuhler MC, Spyker DA, Brooks DE, Dibert KW, *et al*. 2019 Annual report of the American Association of Poison Control Centers’ National Poison Data System (NPDS): 37th annual report. Clin Toxicol (Phila). 2020;58(12):1360–541. 10.1080/15563650.2020.1834219 33305966

[9] Noble MJ, Hedberg K, Hendrickson RG. Acute cannabis toxicity. Clin Toxicol (Phila). 2019;57(8):735–42. 10.1080/15563650.2018.1548708 30676820

[10] Large M, Sharma S, Compton MT, Slade T, Nielssen O. Cannabis use and earlier onset of psychosis: a systematic meta-analysis. Arch Gen Psychiatry. 2011;68(6):555–61. 10.1001/archgenpsychiatry.2011.5 21300939

[11] Gage SH, Hickman M, Zammit S. Association between cannabis and psychosis: epidemiologic evidence. Biol Psychiatry. 2016;79(7):549–56. 10.1016/j.biopsych.2015.08.001 26386480

[12] D’Souza DC, Perry E, MacDougall L, Ammerman Y, Cooper T, Wu Y, *et al*. The psychotomimetic effects of intravenous delta-9-tetrahydrocannabinol in healthy individuals: implications for psychosis. Neuropsychopharmacology. 2004;29(8):1558–72. 10.1038/sj.npp.1300496 15173844

[13] Volkow ND, Swanson JM, Evins AE, DeLisi LE, Meier MH, Gonzalez R, *et al*. Effects of cannabis use on human behavior, including cognition, motivation, and psychosis: a review. JAMA Psychiatry. 2016;73(3):292–7. 10.1001/jamapsychiatry.2015.3278 26842658

[14] Pertwee R. Handbook of cannabis. Oxford: Oxford University Press; 2014.

[15] Voce A, Burns R, Castle D, Calabria B, McKetin R. Is there a discrete negative symptom syndrome in people who use methamphetamine? Compr Psychiatry. 2019;93:27–32. 10.1016/j.comppsych.2019.06.002 31301605

[16] Montague DK, Jarow J, Broderick GA, Dmochowski RR, Heaton JPW, Lue TF, *et al*. American Urological Association guideline on the management of priapism. J Urol. 2003;170(4 Pt 1):1318–24. 10.1097/01.ju.0000087608.07371.ca 14501756

[17] Montgomery S, Sirju K, Bear J, Ganti L, Shivdat J. Recurrent priapism in the setting of cannabis use. J Cannabis Res. 2020;2(1):7. 10.1186/s42238-020-0015-8 PMC7819303 33526129

[18] Evans L, Larsen M, Cox A, Skyrme R. Steroids, drugs and stuttering priapism; the rock-and-roll lifestyle of a 24-year-old man. BMJ Case Rep. 2016. 10.1136/bcr-2015-212448. PMC4735370 26822610

[19] Tran QT, Wallace RA, Sim EHA. Priapism, ecstasy, and marijuana: is there a connection? Adv Urol. 2008;2008:193694. 10.1155/2008/193694 PMC2441841 18604290

[20] Melis MR, Succu S, Sanna F, Melis T, Mascia MS, Enguehard-Gueiffier C, *et al*. PIP3EA and PD-168077, two selective dopamine D4 receptor agonists, induce penile erection in male rats: site and mechanism of action in the brain. Eur J Neurosci. 2006;24(7):2021–30. 10.1111/j.1460-9568.2006.05043.x 17067298

[21] Dean RC, Lue TF. Physiology of penile erection and pathophysiology of erectile dysfunction. Urol Clin North Am. 2005;32(4):379–95. 10.1016/j.ucl.2005.08.007 PMC1351051 16291031

[22] Succu S, Mascia MS, Sanna F, Melis T, Argiolas A, Melis MR. The cannabinoid CB1 receptor antagonist SR 141716A induces penile erection by increasing extra-cellular glutamic acid in the paraventricular nucleus of male rats. Behav Brain Res. 2006;169(2):274–81. 10.1016/j.bbr.2006.01.017 16516985

